# Correlational Analysis of Serum Metabolites and Cognitive Function in Moyamoya Disease

**DOI:** 10.1002/alz70856_100640

**Published:** 2025-12-24

**Authors:** Jun Shen, Sisi Peng, Juan Du, Zhengxin Liu, Mingxuan Lv, Yu Duan, Wenshi Wei

**Affiliations:** ^1^ Changhai hosptial, Naval Medical University, Shanghai, Shanghai, China; ^2^ Huadong Hospital of Fudan University, Shanghai, Shanghai, China; ^3^ Zhongnan Hospital of Wuhan University, Wuhan, Hubei, China

## Abstract

**Background:**

Cognitive dysfunction is a common symptom in moyamoya disease (MMD). However, the mechanisms underlying this impairment, particularly the changes in serum metabolites, have not been thoroughly investigated. The primary objective of this study was to utilize untargeted metabolomics technology to analyze serum metabolic profiles in MMD patients and to explore the potential relationship between metabolic alterations and cognitive function.

**Method:**

Thirty patients with MMD and ten healthy controls were enrolled in our study. Cognitive assessment composing Montreal Cognitive Assessment (MoCA) and Hopkins Verbal Learning Test‐Revised (HVLT‐R) were used in this study to appraise general cognition and specific cognitive domains, including immediate recall, delayed recall, and delayed recognition. Serum samples were collected for metabolomics analysis that through using liquid chromatography–tandem mass spectrometry (LC‐MS/MS) to quantify and identify metabolomics. Differentially expressed metabolites were identified based on fold changes and statistical significance. Additionally, correlation analyses were performed to evaluate the associations between identified differential metabolites and cognitive function.

**Result:**

Our results found that overall cognitive function and the subdomains of cognitive function include immediate memory, delayed recall, and recognition in MMD patients were significantly impaired. The untargeted metabolomics analysis identified 142 significantly altered metabolites in the serum of MMD patients compared to controls. Of these, 22 metabolites were downregulated, while 120 metabolites were upregulated. We identified significant alterations in bile acid, amino acid, peptide, and purine metabolism, along with changes in lipid metabolism, inflammation, hormonal regulation, and protein metabolism in the MMD group, suggesting a complex metabolic dysregulation in MMD. Furthermore, correlation analysis indicated that the metabolomic changes in MMD patients were strongly associated with cognitive dysfunction, implying their potential involvement in the pathophysiology of cognitive impairment.

**Conclusion:**

In conclusion, our study provides evidence that cognitive dysfunction in MMD are associated with significant metabolic changes. The altered serum metabolites highlight the needs to comprehensively understand and manage cognitive dysfunction in MMD patients.

